# Supplementary material for: Heterogeneous dissociation process of truncated RNAs by oligomerized Vasa helicase
Source: Commun Biol. 2021 Dec 10;4:1386. doi: 10.1038/s42003-021-02918-0 (PMC8664846; doi:10.1038/s42003-021-02918-0)
Supplement: Supplementary file 2 — Supplementary Information [file 42003_2021_2918_MOESM2_ESM.pdf]

Supplementary Information

Heterogeneous dissociation process of truncated RNAs by oligomerized Vasa helicase

Yoshimi Kinoshita[1], Ryo Murakami[1], Nao Muto[1], Shintaroh Kubo[2], Ryo Iizuka[1] and Sotaro Uemura[1#]

[1] Department of Biological Sciences, Graduate School of Science, The University of Tokyo, Tokyo, Japan

[2] Department of Biophysics, Graduate School of Science, Kyoto University Kyoto, Japan

# Corresponding author.

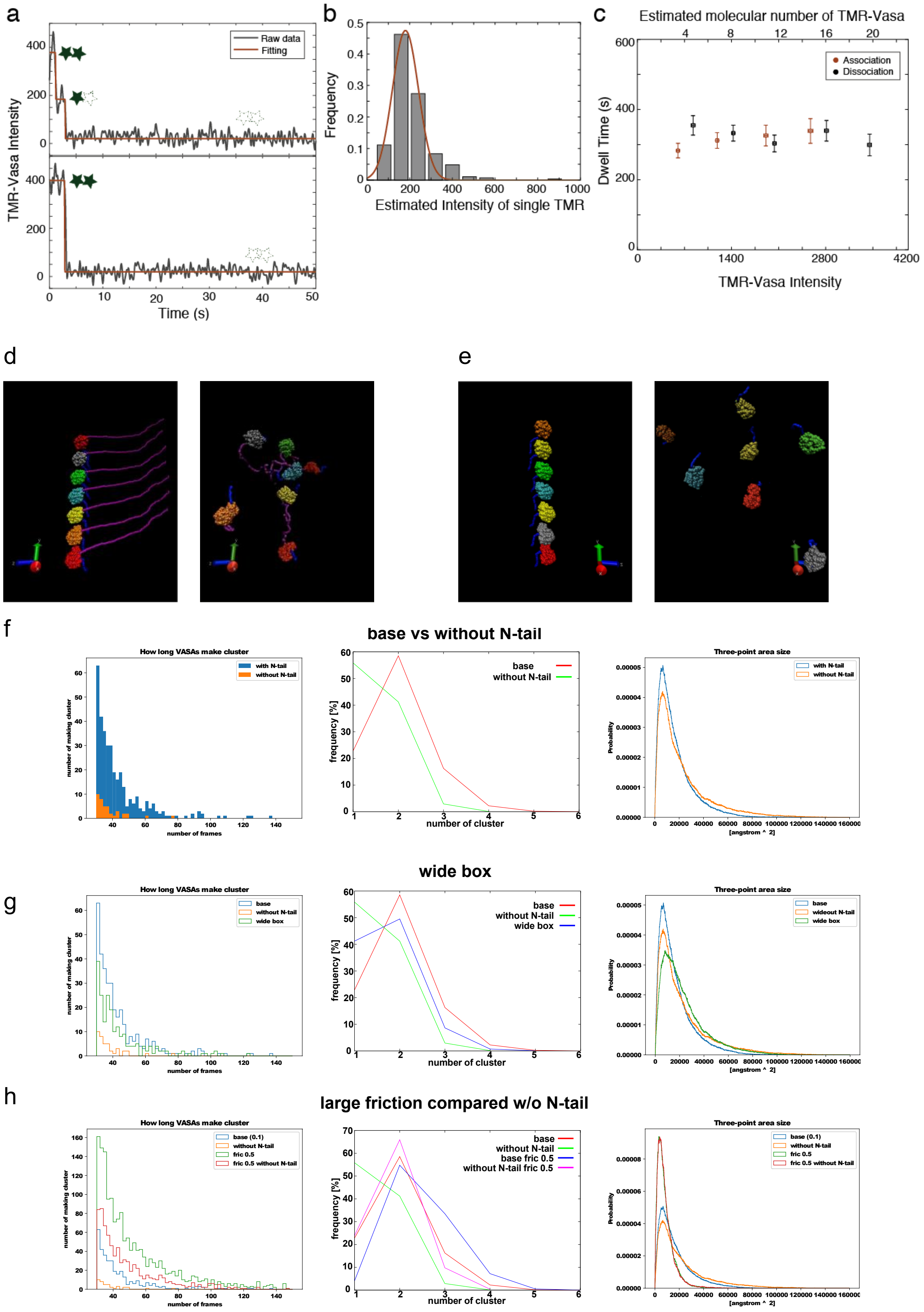
a
Raw data
Fitting
TMR-Vasa Intensity
Time (s)
b
Frequency
Estimated Intensity of single TMR
c
Estimated molecular number of TMR-Vasa
Association
Dissociation
Dwell Time (s)
TMR-Vasa Intensity
d
e
f
base vs without N-tail
How long VASAs make cluster
with N-tail
without N-tail
number of making cluster
number of frames
base
without N-tail
frequency [%]
number of cluster
Three-point area size
Probability
[angstrom ^ 2]
g
wide box
base
without N-tail
wide box
wideout N-tail
h
large friction compared w/o N-tail
base (0.1)
fric 0.5
fric 0.5 without N-tail
base fric 0.5
without N-tail fric 0.5

Figure S1 Additional information about Single molecular intensity of TMR-Vasa oligomerization on Figure 1. (**a**) A representative photobleaching trace of TMR-Vasa. Red lines show the identified bleaching stages, where were shown in arrows. Single- and double-bleaching stages were 67 % and 33 %, respectively. (**b**) Gaussian distribution of the single TMR intensity (178.2 ± 58.6, mean ± S.D.) labeled on Vasa calculated from the photobleaching results (R^2=0.973, N=288). (**c**) The oligomerized molecule number of Vasa was independent of the dwell time before Vasa association (red) or dissociation (black) ($p > 0.05$; Welch's t-test). Error bars are mean ± S.E.M. (N>30 in each bar). (**d-g**) Verification of Vasa clusters in coarse-grained molecular dynamic simulation. (**d, e**) The initial states (left) and the typical interacting states (right) in seven WT-Vasa (**d**) and dN-Vasa (**e**) molecules. (**f**) Comparison between WT-Vasa ('base') and dN-Vasa ('without N-tail'). (**g**) Comparison between WT-Vasa ('base', 'wide box') and dN-Vasa ('without N-tail'). (**h**) Comparison between WT-Vasa ('base', 'base fric 0.5' in large friction) and dN-Vasa ('without N-tail', 'without N-tail fric 0.5). (**f-h**) Left: the frequency of successive frames of intermolecular interaction. Center: the number of Vasa molecules that interact with a given Vasa to compose the cluster. Right: the molecular density calculated from the centre of mass gravity of any three Vasa molecules.

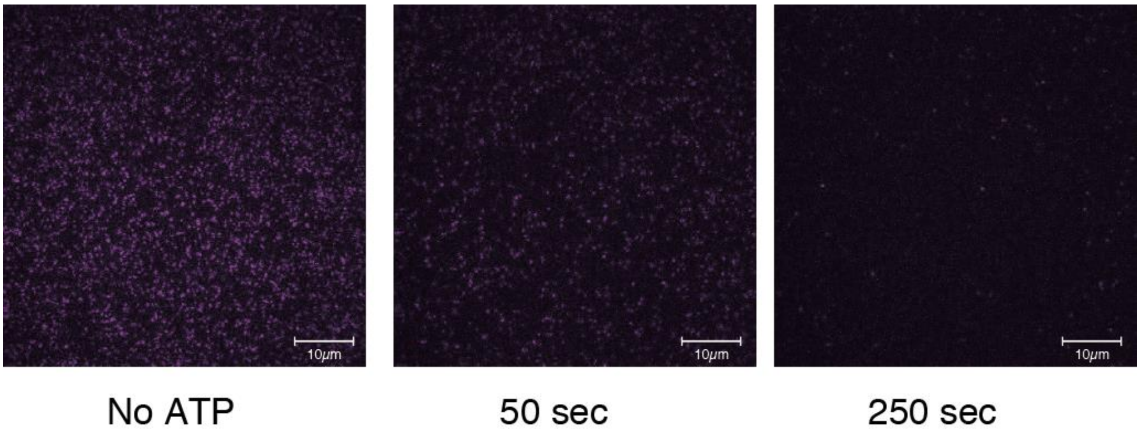

Figure S2 The subsequent fluorescent images (60.4 x 60.4 μm^2) of target RNA incubated with Siwi-piRISC, Vasa and 1 mM ATP, shown in Fig. 2b. Scale bars represent 10 μm.

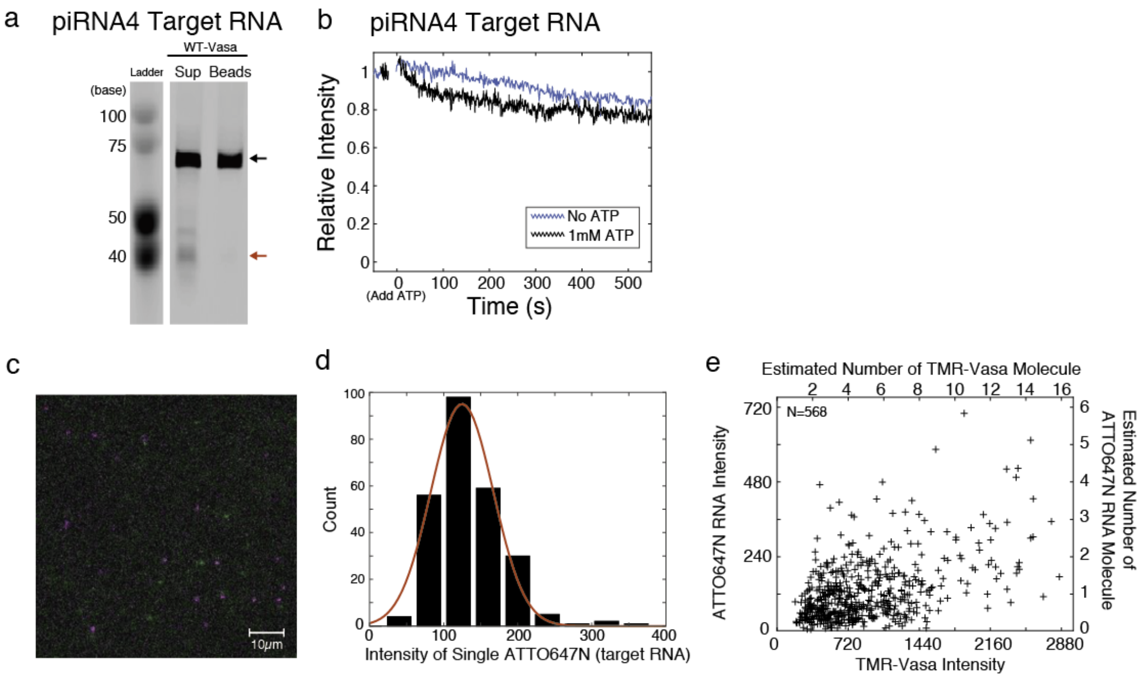

Figure S3 Additional information of target RNA, its dissociation and overlapping with Vasa on Figure 3. ~~Two-coloured molecular measurements.~~ (**a**) RNA cleavage assay of piRNA4-target RNA. In the presence of Siwi, Vasa and ATP, target RNA was cleaved and dissociated from Siwi-piRISC in bulk. Black and red arrows indicate the target RNA and cleaved RNA, respectively. Full gel image is shown in Figure S6a. (**b**) The relative intensity decay of target RNA after adding 0 (blue) or 1mM (black) ATP with Vasa in a certain (27.6 x 27.6 μm^2) region. (**c**) The fluorescent image (73.8 x 73.8 μm^2) of target RNA (magenta) and non-localized Vasa (green) in the absence of piRISC-Siwi. Scale bar represents 10 μm. (**d**) The distribution of fluorescent intensity of single ATTO647N-labeled piRNA4-target RNA. The single ATTO647N intensity was calculated to be 124 ± 42.1 (mean ± S.D., R^2=0.983, N=257). (**e**) Scatter plot of fluorescent intensities (or the estimated molecular numbers) at the spots where both Vasa and RNA are localized. The molecular numbers of Vasa and RNA were calculated from the averaged intensity of respective single fluorescent dye. The correlation coefficient was 0.689.

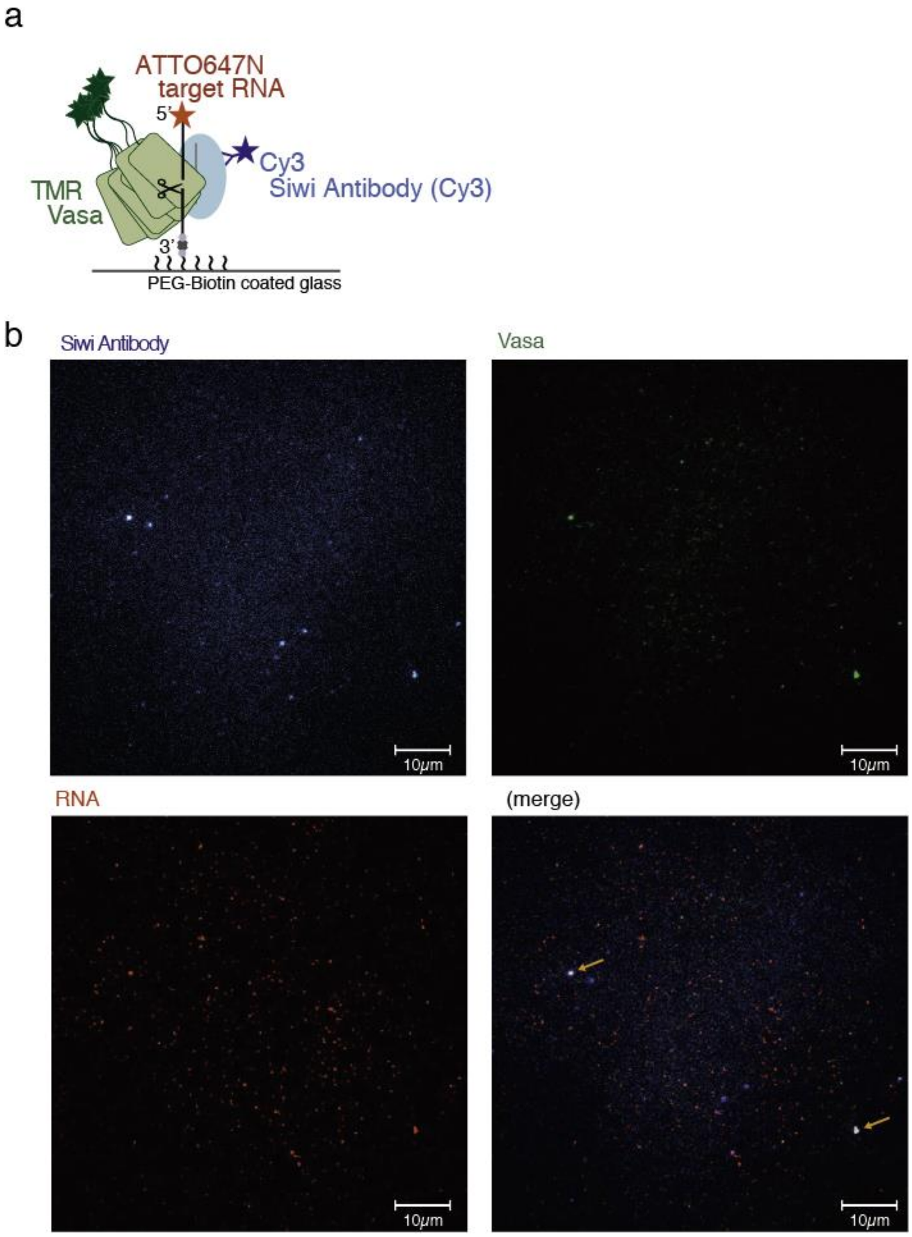

Figure S4 ~~Complex formation~~Reconstruction of Siwi-piRISC with RNA and Vasa. (**a**) Schematic representation of RNA with Vasa and Siwi. (**b**) The fluorescent images (73.8 x 73.8 μm^2) of target RNA (ATTO647N, red) colocalized with the oligomerized WT-Vasa (TMR, green) and anti-Siwi antibody (Alexa488, blue). Arrows show the overlap points. Scale bars represent 10 μm.

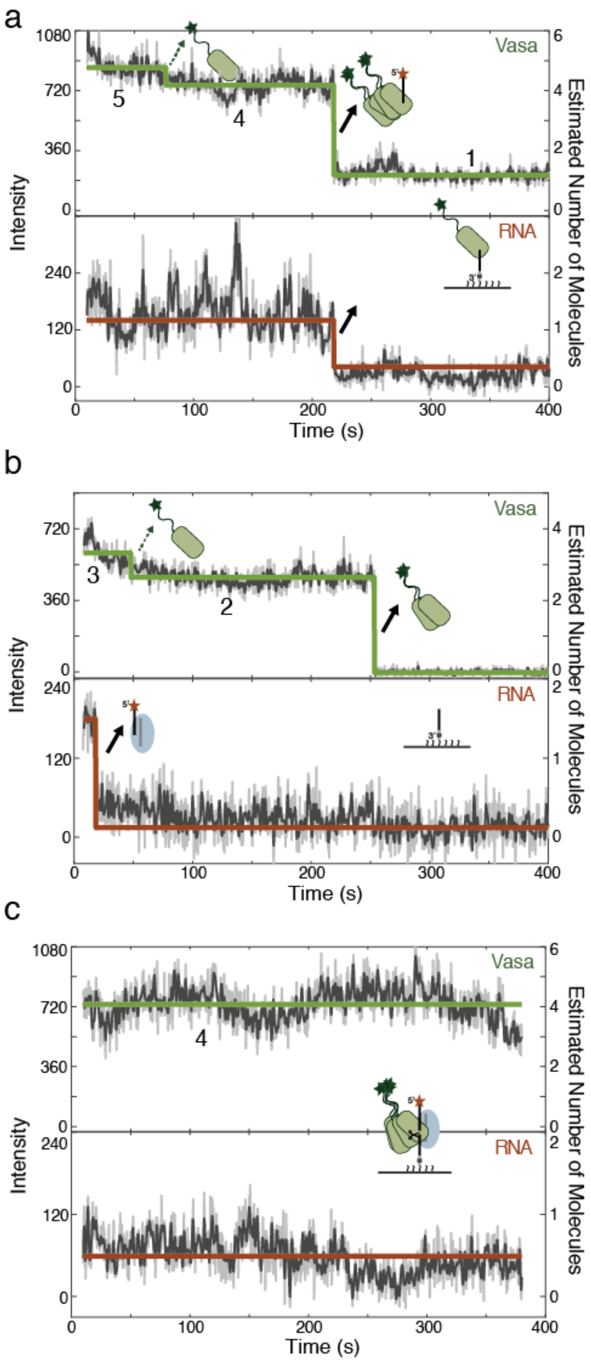

Figure S5 Additional examples of time-trace intensities of the colocalized Vasa (upper, green) and cleaved RNA (lower, red) dissociating after adding 1 mM ATP, referred to (Fig 3**c**-**e**). (**a**) Vasa and RNA dissociated simultaneously at last. (**b**) Vasa partially dissociated independently of RNA dissociation. Partial dissociation of Vasa was observed. (**c**) Both RNA and Vasa kept binding. The molecular numbers of Vasa and RNA were calculated from the averaged intensity of respective single fluorescent dye.

a

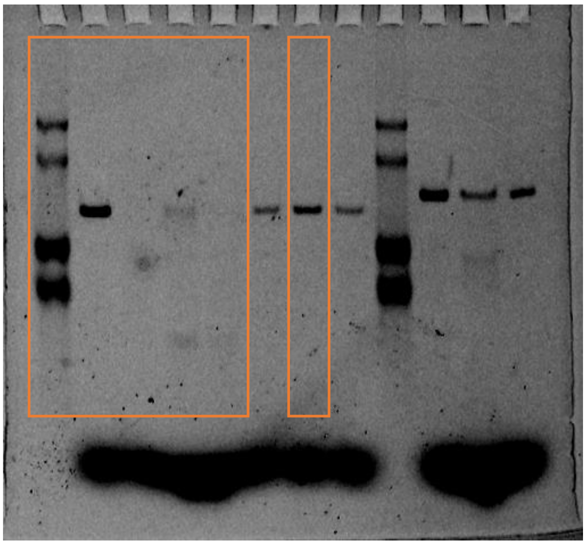

b

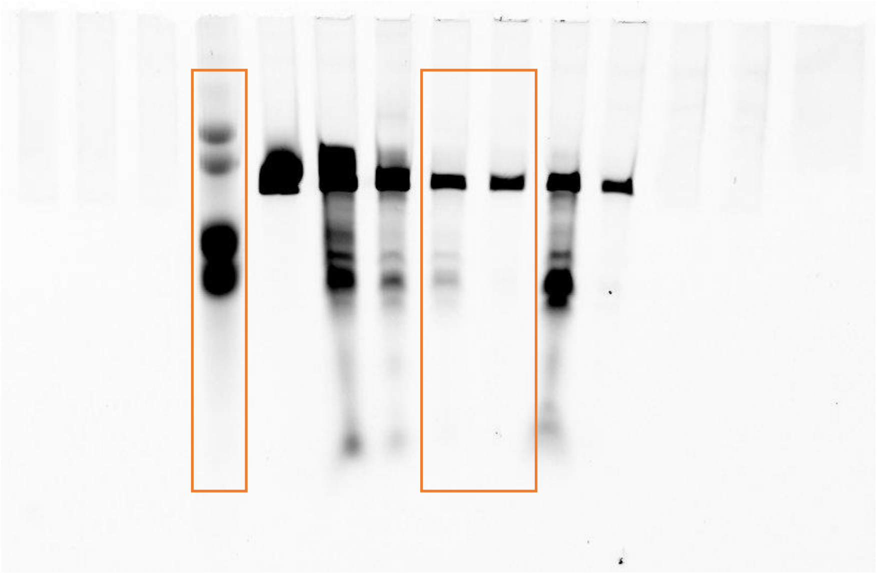

Figure S6 Full gel images related to Figure 2a (**a**) and S3a (**b**). The squares shown in the source images indicate the cropped region.

Supplementary Notes

Coarse-grained molecular dynamic (MD) simulation of Vasa

For the reference structure, we used *Bombyx mori* Vasa structure form[1] (PDB ID: 4D25). This structural model had some missing residues, so we used Clustal Omega for alignment the full-length sequence with 4D25 model[2] (provided from EMBL-EBI). Then, we used MODELLER for making homology model[3].

Based on the atomic structure of Vasa, coarse-grained MD simulation was performed using the CafeMol 2.1[4]. In the coarse-grained model, each amino acid was represented as a single bead located at its Cα position. For getting dynamics, we used the energy functions AICG2+, excluded volume, and electrostatic interaction. In the AICG2+, the original reference structure was assumed as the most stable structure, and parameters could be modified to represent the interactions in the reference structure. Of note, the region we didn't have reference structural information treated flexible because their reference conformations were unreliable.

We performed seven WT-Vasa or dN-Vasa molecules coarse-grained MD simulations at 10 times for each condition (Fig. S1d and e). Each MD simulation took 10^8 MD steps. Note, one MD step roughly corresponds to ~1ps but the time mapping was a little inaccurate. The MD simulations were conducted by the underdamped Langevin dynamics at 300K temperature. We add the box potential for control concentration of Vasa. We set the rectangular box size of 16, 75, and 75 nm for the x, y, and z-axis, respectively. We set the friction coefficient to 0.1 (CafeMol unit), and default values in CafeMol were used for other parameters.

We defined that Vasa make the clusters when the electrostatic interaction was working continuously for more than 30 frames. Vasa made clusters for long time frames. The deletion of N-IDR of Vasa resulted in the formation of clusters less frequently and their duration was significantly shorter (Fig. S1f, left). We next investigated how many Vasa molecules compose the clusters. Vasa can make the clusters with two or more Vasa molecules. Even some dN-Vasa could form the defined clusters, but the fraction that interacted with other dN-Vasa molecules was quite lower (Fig. S1f, center). We analyzed the molecular density calculated from the center of mass gravity of any three molecules at each frame. Vasa could make the dense triangle formation rather than dN-Vasa (Fig. S1f, right).

When the box size of the x-axis was extended to 75nm, the collision frequency of Vasa was definitely decreased, however, the frequency of molecular interactions for longer than 60 frames did not differ significantly (Fig. S1g). This suggests that Vasa can be stabilized locally in the cluster formation independent of the observed volume.

Furthermore, we also examined the degree of cluster formation by increasing the friction coefficient from 0.1 to 0.5. Even in the absence of N-IDR of Vasa, the frequency of the defined cluster formation increased with an increase of viscosity. To construct a large cluster consisting of three or more Vasa molecules, the N-IDR of Vasa is required (Fig. S1h). These results indicate that N-IDRs of Vasa trigger the large clusters via the electrostatic interaction and stabilize in a dense state.

Supplementary References

1. Xiol, J. et al. RNA clamping by Vasa assembles a piRNA amplifier complex on transposon transcripts. *Cell* **157**, 1698-1711 (2014).
2. Sievers, F. et al. Fast, scalable generation of high-quality protein multiple sequence alignments using Clustal Omega. *Mol. Syst. Biol.* 7:539 (2011).
3. Fiser, A. and Sali A. Modeller: generation and refinement of homology-based protein structure models. *Methods Enzymol.* 374: 461-491 (2003).
4. Kenzaki, H. et al. CafeMol: a coarse-grained biomolecular simulator for simulating proteins at work. *J. Chem. Theory Comput.* 7: 1979-1989 (2011).
