# Supplementary material for: Heterogeneous dissociation process of truncated RNAs by oligomerized Vasa helicase
Source: Commun Biol. 2021 Dec 10;4:1386. doi: 10.1038/s42003-021-02918-0 (PMC8664846; doi:10.1038/s42003-021-02918-0)
Supplement: Supplementary file 3 — Description of Additional Supplementary Files [file 42003_2021_2918_MOESM3_ESM.pdf]

# Description of Additional Supplementary Files

**File name:** Supplementary Data 1.
**Description:** Source data for graphs and charts generated for the main figures and supplementary figures.
