# Supplementary material for: Heterogeneous dissociation process of truncated RNAs by oligomerized Vasa helicase
Source: Commun Biol. 2021 Dec 10;4:1386. doi: 10.1038/s42003-021-02918-0 (PMC8664846; doi:10.1038/s42003-021-02918-0)
Supplement: Supplementary file 5 — Reporting Summary [file 42003_2021_2918_MOESM5_ESM.pdf]

nature research

Corresponding author(s): Sotaro Uemura

Last updated by author(s): Oct 27, 2021

Reporting Summary

Nature Research wishes to improve the reproducibility of the work that we publish. This form provides structure for consistency and transparency in reporting. For further information on Nature Research policies, see our Editorial Policies and the Editorial Policy Checklist.

Statistics

For all statistical analyses, confirm that the following items are present in the figure legend, table legend, main text, or Methods section.

n/a	Confirmed	
☐	☒	The exact sample size (n) for each experimental group/condition, given as a discrete number and unit of measurement
☐	☒	A statement on whether measurements were taken from distinct samples or whether the same sample was measured repeatedly
☒	☐	The statistical test(s) used AND whether they are one- or two-sided<br>*Only common tests should be described solely by name; describe more complex techniques in the Methods section.*
☒	☐	A description of all covariates tested
☒	☐	A description of any assumptions or corrections, such as tests of normality and adjustment for multiple comparisons
☐	☒	A full description of the statistical parameters including central tendency (e.g. means) or other basic estimates (e.g. regression coefficient) AND variation (e.g. standard deviation) or associated estimates of uncertainty (e.g. confidence intervals)
☒	☐	For null hypothesis testing, the test statistic (e.g. F, t, r) with confidence intervals, effect sizes, degrees of freedom and P value noted<br>*Give P values as exact values whenever suitable.*
☒	☐	For Bayesian analysis, information on the choice of priors and Markov chain Monte Carlo settings
☒	☐	For hierarchical and complex designs, identification of the appropriate level for tests and full reporting of outcomes
☒	☐	Estimates of effect sizes (e.g. Cohen's d, Pearson's r), indicating how they were calculated

Our web collection on statistics for biologists contains articles on many of the points above.

Software and code

Policy information about availability of computer code

Data collection	Fluorescence images, except for immunostaining images, were obtained using a home-made microscope system, which was controlled by Micro-Manager software. Immunostaining images were acquired using LSM800 with AiryScan, controlled by ZEISS ZEN imaging software.
Data analysis	Fluorescent images were processed by ImageJ/Fiji (NIH). The transitions of intensities were detected based on a hidden Markov Model using MATLAB R2020b (MathWorks).

For manuscripts utilizing custom algorithms or software that are central to the research but not yet described in published literature, software must be made available to editors and reviewers. We strongly encourage code deposition in a community repository (e.g. GitHub). See the Nature Research guidelines for submitting code & software for further information.

Data

Policy information about availability of data

All manuscripts must include a data availability statement. This statement should provide the following information, where applicable:

- Accession codes, unique identifiers, or web links for publicly available datasets
- A list of figures that have associated raw data
- A description of any restrictions on data availability

The authors declare that the data are available within the paper and its Supplementary Data files.

April 2020

Field-specific reporting

Please select the one below that is the best fit for your research. If you are not sure, read the appropriate sections before making your selection.

☒ Life sciences ☐ Behavioural & social sciences ☐ Ecological, evolutionary & environmental sciences

For a reference copy of the document with all sections, see nature.com/documents/nr-reporting-summary-flat.pdf

Life sciences study design

All studies must disclose on these points even when the disclosure is negative.

Sample size	Sample sizes were not predetermined based on statistical methods, but were chosen according to the standards of the field (at least three independent biological replicates for each condition), which generated a sufficient number of single-molecule trajectories and gave sufficient statistics for the effect sizes of interest.
Data exclusions	No data was excluded.
Replication	All experiments were taken more than three times.
Randomization	Quantitative analysis of experiments were done using randomly selected images.
Blinding	The investigators were not blinded because no group allocation was performed.

Reporting for specific materials, systems and methods

We require information from authors about some types of materials, experimental systems and methods used in many studies. Here, indicate whether each material, system or method listed is relevant to your study. If you are not sure if a list item applies to your research, read the appropriate section before selecting a response.

Materials & experimental systems

n/a	Involved in the study	
☐	☒	Antibodies
☐	☒	Eukaryotic cell lines
☒	☐	Palaeontology and archaeology
☒	☐	Animals and other organisms
☒	☐	Human research participants
☒	☐	Clinical data
☒	☐	Dual use research of concern

Methods

n/a	Involved in the study	
☒	☐	ChIP-seq
☒	☐	Flow cytometry
☒	☐	MRI-based neuroimaging

Antibodies

Antibodies used	Anti-FLAG M2 antibody, Sigma (#2220); Anti-Siwi antibody IgG2a, kindly gifted from Mikiko C. Siomi lab (The University of Tokyo, Nishida et al. Cell Reports 2015); Alexa Fluor 488/555 goat anti-mouse IgG1/IgG2a antibodies, Invitrogen (#A28175, #A28180, respectively).
Validation	All antibodies were validated by immunofluorescence using tagged Vasa or endogeneous Siwi.

Eukaryotic cell lines

Policy information about cell lines

Cell line source(s)	BmN4 cells used in this study were kindly provided by Mikiko C. Siomi lab (The University of Tokyo) derived from National Institute of Agrobiological Sciences (NIAS).
Authentication	We think that BmN4 cells used have been authenticated, because BmN4 were gifted from Mikiko C. Siomi lab derived from National Institute of Agrobiological Sciences (NIAS).
Mycoplasma contamination	BmN4 cells were not tested for mycoplasma contamination.
Commonly misidentified lines (See ICLAC register)	BmN4 cells are not misidentified cell lines.
